# Endectocide-treated cattle for malaria control: A coupled entomological-epidemiological model

**DOI:** 10.1016/j.parepi.2015.12.001

**Published:** 2015-12-31

**Authors:** Laith Yakob

**Affiliations:** Department of Disease Control, Faculty of Infectious and Tropical Diseases, London School of Hygiene and Tropical Medicine, Keppel Street, London WC1E 7HT, United Kingdom

**Keywords:** Systemic insecticide, Topical insecticide, Anopheles, Zoophilic vector, Mathematical model, Mosquito control

## Abstract

The malaria vector landscape is dynamic and dependence on indoor control tools has drastically affected both species compositions and local mosquito biting behaviours. In the advent of spreading behavioural resilience and physiological resistance to insecticidal nets and house spray, approaches to target more zoophilic, outdoor-biting vectors are being sought with increased urgency. Endectocides are insecticides applied to hosts which are taken up by the vectors during biting, and recent field assessments have demonstrated favourable results of cattle treated with ivermectin, diflubenzuron, eprinomectin and fipronil. Models were constructed to account for the modern, diverse vector feeding behaviours and assess their role in shaping malaria transmission and control with cattle-treated endectocides. Efficacy of this novel approach to malaria control is shown to be strongly dependent not only on intrinsic host preferences of the vector but also on how this preference is augmented by variation in the encounter rates with alternative blood-hosts. Ecological scenarios are presented whereby endectocides used on cattle yield equivalent, and in some cases improved, efficacy over nets and spray in controlling malaria transmission. Interactions between mosquito biting behaviours and relative availabilities of alternative blood-host species have largely been neglected in malaria programmatic strategy but will increasingly underlie sustaining the successes of vector control initiatives.

## 1 Introduction

Within both control and elimination settings, the primary means of malaria reduction has been through vector management with chemical insecticides (Bhatt et al., 2015). Widespread resistance among numerous disparate populations of vectors has raised serious concern over the sustainability of thus far successful disease control campaigns. Lessons learned during the era of dwindling vector control efficacy include the necessity for optimising control strategy of any new insecticidal approaches to controlling malaria that come to market in order to maximise, and ideally prolong, the benefit achieved with these precious few resources (Mnzava et al., 2015). The current study sought to assess an endectocidal approach to kill malaria vectors and to identify the ecological conditions that would optimise its application.

Endectocides are insecticides applied directly to hosts to kill blood-feeding arthropods. They can either be applied topically, parenterally or they can be ingested by the host resulting in their dissemination through the circulatory system. A forerunner chemical for this strategy is ivermectin which antagonises the insect-specific glutamate-gated chloride neuromuscular transmission channels (Kane et al., 2000). Importantly, this mode of action simultaneously impedes any major toxic effects in treated mammals — because they lack this channel (Bloomquist, 2003) - while protecting its efficacy from inadvertent cross-resistance with any of the insecticides currently used as vector control mainstays (Foy et al., 2011). Although its primary application in human disease control is within mass drug administration campaigns for onchocerciasis, empirical evidence is mounting for ivermectin effectively controlling tsetse fly vectors of African trypanosomiasis (Pooda et al., 2013) as well as anopheline malaria vectors (Fritz et al., 2009). However, theoretical studies are comparatively scant and require considerable development for this approach to be strategically assessed in the context of disease control — both as a stand-alone method and as a component of integrated vector management.

Expanding ideas first raised by Wilson (1993), Sylla et al. (2010) developed an age-structured model of *Anopheles gambiae* and assessed treating humans with ivermectin having the dual effect of reducing vector densities (through instantaneous death) while also skewing mosquito demography towards a younger population (through shortened lifespan resulting from sub-lethal dose exposure). Simulations of these authors demonstrated a 90% reduction in the malaria reproduction number, intuitively showing that maximal gains were achieved when treatment coverage and frequency was highest (Sylla et al., 2010). A key disadvantage to this general strategy was the short half-life of typical ivermectin doses in humans resulting in rapidly attenuating effects. However, subsequent to this initial work, higher dose formulations have been optimised for increased longevity in cattle and new chemicals have proven effective additions to the endectocide arsenal.

Diflubenzuron, eprinomectin and fipronil have all shown promise in initial trials in the control of sand fly vectors of leishmaniasis (Ingenloff et al., 2013). Very recently, the first field demonstration was published of the effective treatment of cattle with these chemicals to kill *A.* *gambiae* sensu lato (Poche et al., 2015). These pose interesting developments in the context of the current malaria vector landscape whereby compositional changes have favoured dominance of exophilic species (Mwangangi et al., 2013) or vectors that have demonstrated increased plasticity in their biting behaviours (Reddy et al., 2011). Understanding the biting behaviour of disease vectors in terms of the distribution of bites among different host species has become a higher priority following these developments; and, in the current study, a novel framework is presented to improve how this behaviour is captured by models to assess the epidemiological impact of applying insecticides to different blood host species.

## 2 Methods

For demonstrating the consequences of how mosquitoes distribute their bites among two alternative host species, the benchmark is to assume that the proportion of bites on either species is directly proportional to their relative availability. In other words, doubling the availability of humans relative to all blood host species doubles the proportion of bites that befall humans; and this is the underlying assumption of models that have previously explored the role of bites on non-human hosts in malaria epidemiology. In reality, arthropods have never demonstrated this linear response to resource availability (Jeschke et al., 2004). A parsimonious yet flexible function that allows for different forms of resource utilisation is:

(1) $$p=\frac{Q}{Q+\alpha {\left(1-Q\right)}^{\beta }.}$$

**Fig. 1:**
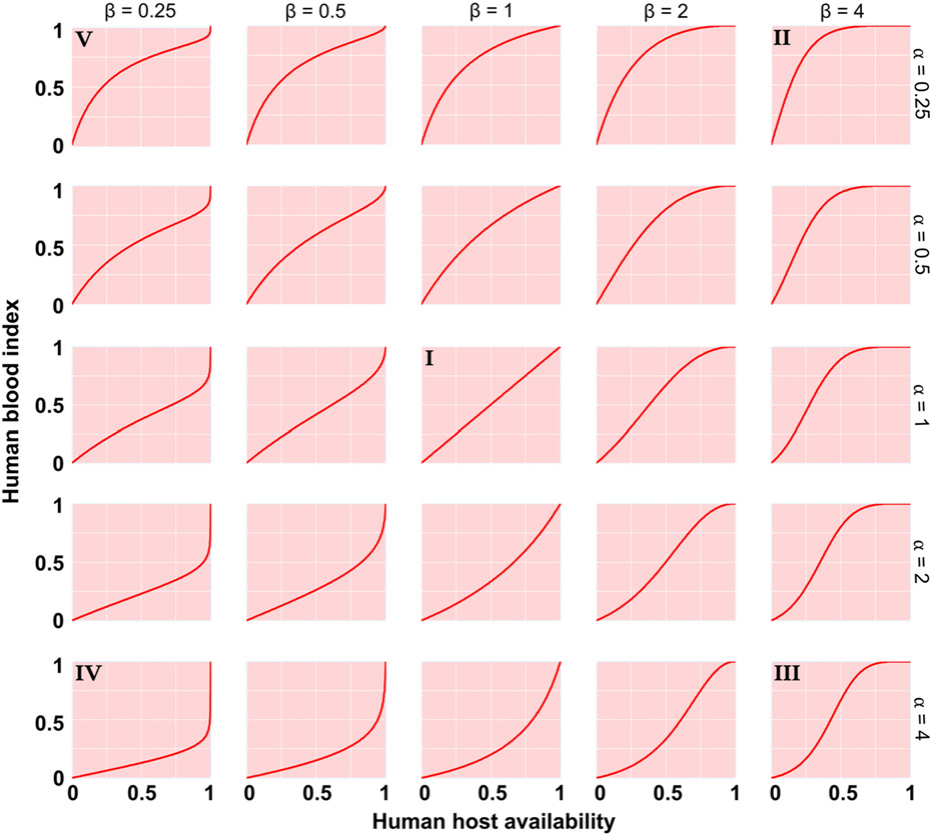
The human blood index of a disease vector across varying levels of human host availability (relative to all potential blood sources) as augmented by different biting behaviours. Distinct qualitative forms of vector behaviour (denoted ‘I’ to ‘V’) are shaped by parameters *α* and *β* as described in Eq. 1.

Where, *p* is the proportion of all blood meals that are derived from the species of interest; Garrett-Jones (1964) described the anthropocentric equivalent as the 'human blood index' (HBI); *Q* is the availability of the host species of interest relative to all potential blood hosts; *α* and *β* are parameters that shape the behavioural response. Fig. 1 demonstrates the effects that *α* and *β* have on the functional response with this new formulation. Five qualitatively distinct functional forms are represented in the figure and they each have different implications for vector biting behaviour. In keeping with the functional response nomenclature of Holling, (1959), but expanding it to account for a more diverse set of biological responses, the Types I–V vector responses can be interpreted as follows:

• Type I describes an indiscriminate mosquito; or, biting that is directly proportional to the relative availabilities of host species
• Type II describes an anthropophilic mosquito with HBI nearing unity even when alternative hosts are equally available
• Type III describes a mosquito demonstrating a switch in preference beyond a certain threshold in relative availabilities
• Type IV describes a zoophilic mosquito uninclined to bite humans unless they constitute all but the only available hosts
• Type V describes a negative prey-switching (Abrams et al., 1993) analogue; beyond a threshold, the host exhibits defensive behaviour.

As is shown in Fig. 1, for the same availability of humans relative to cattle, a drastically different proportion of mosquito bites on humans can arise with this new formulation. The exceptions obviously being when humans are entirely unavailable or when they represent the only blood source. In order to demonstrate transparently the impact of this neglected aspect of vector behavioural ecology, this function is integrated into a simple epidemiological model of malaria transmission:

(2) $$\frac{\mathrm{dI}}{\mathrm{dt}}=mp_{H}\beta_{H}\mathrm{SZ}-\left(\varepsilon +\mu_{H}\right)I\;$$

(3) $$\frac{\mathrm{dR}}{\mathrm{dt}}=\mathrm{\varepsilon I}+\mathrm{\kappa A}-\left(\mathrm{\theta m}p_{H}\beta_{H}Z+\tau +\mu_{H}\right)R\;$$

(4) $$\frac{\mathrm{dA}}{\mathrm{dt}}=\theta mp_{H}\beta_{H}\mathrm{RZ}-\left(\kappa +\mu_{H}\right)A\;$$

(5) $$\frac{\mathrm{dY}}{\mathrm{dt}}=p_{H}\beta_{Z}\left(I+\mathrm{\sigma A}\right)X-\left({\zeta +\mu }_{V}\right)Y\;$$

(6) $$\frac{\mathrm{dZ}}{\mathrm{dt}}=\mathrm{\zeta Y}-\mu_{V}Z.\;$$

Where, ‘*S*’usceptible hosts become ‘*I*’nfected following a bite from an infectious mosquito, ‘*Z*’. Infected hosts can benefit from some level of temporary immunity (1 − *θ*, where 0 ≤ *θ* ≤ 1) before reversion to fully susceptible at rate ‘*τ*’. However, when re-infected during this ‘*R*’ecovered state, hosts can become ‘*A*’symptomatically infected. Asymptomatic infection may have different transmission potential to vectors (*σ*), and is itself a temporary state after which individuals revert to the recovered category at rate ‘*κ*’. Although human mortality is included, a constant human population is assumed whereby births offset deaths and so *S* = 1 − (*I* + *R* + *A*). Susceptible vectors (*X*) become infected but not yet infectious (*Y*) following a bite on an infectious host, and after the extrinsic incubation period (1/*ζ*), become infectious (*Z*). Vectors typically outnumber hosts and, following convention, the ratio of vector-to-hosts is denoted *m*. Here, a stable vector population is assumed whereby births are set to balance deaths (μ_V_) and so *X* = 1 − (*Y* + *Z*). Parameter values and literature sources are detailed in Table 1.

**Table 1:** Model parameter definitions, values and sources.

	Definition	Value [cited literature]
*b*_*VH*_	Transmission coefficient (vectors➔hosts) = bite rate × transmission probability	0.1 = 1/3 × 0.3 (Rickman et al., 1990)
*b*_*HV*_	Transmission coefficient (hosts➔vectors) = bite rate × transmission probability	0.007 = 1/3 × 0.02 (Bonnet et al., 2003)
ε	Clearance rate of symptomatic infection	1/200 (Falk et al., 2006)
κ	Clearance rate of asymptomatic infection	1/200 (Falk et al., 2006)
θ	Asymptomatic secondary infection rate	0.5 (assumed)
τ	Full susceptibility reversion rate	1/1000 (Doolan et al., 2009)
μ_H_	Birth and death rate of humans (i.e., stable population)	1/21,900 (assumed)
μ	Birth (or maturation) and death rate of vectors (i.e., stable population)	1/10 (Ermert et al., 2011)
σ	Adjustment factor for asymptomatic transmissibility to vector	0.25 (Okell et al., 2012)
ζ	Rate of parasite development within vector	1/10 (Blanford et al., 2013)

These equations are used to explore the role that this aspect of mosquito ecology has in augmenting the outcomes anticipated from insecticidal control of adult vectors. Chemical control is well known to have multiplicative effects on anopheline vectorial capacity. Here, these consist of i) a reduction in bites per human host; ii) and an increased mosquito mortality rate associated with insecticidal contact during human host feeding; or iii) increased mosquito mortality rate associated with insecticidal contact during cattle host feeding. This third option is explored in light of the recent developments in systemic (as well as topical) insecticides used in cattle to reduce malaria transmission in humans.

## 3 Results

**Fig. 2:**
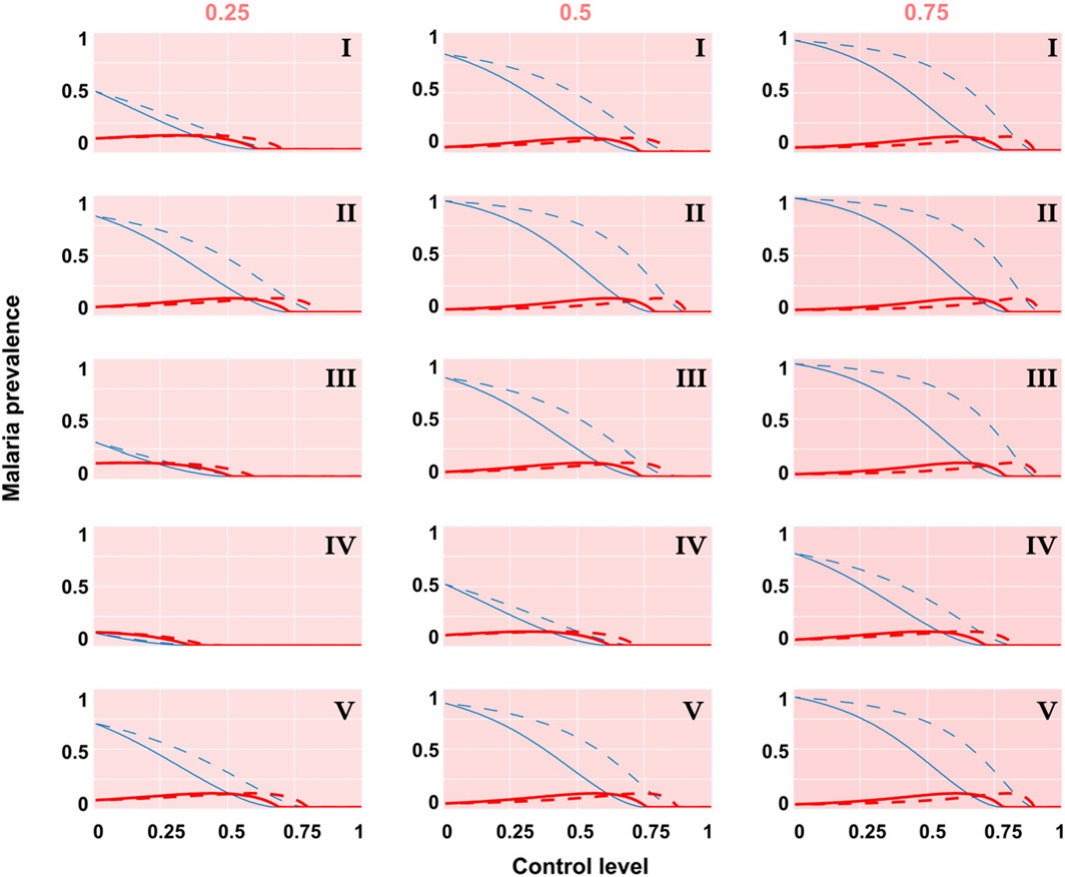
Malaria prevalence (asymptomatic in blue; symptomatic in red) following human-targeted chemical control with a repellent (dashed lines) versus a mosquitocidal product (solid lines). Control efficacy is shown across the qualitatively distinct mosquito biting ecologies (top right of sub-plots) and across a range of the human availability relative to all blood-hosts (top of sub-plot columns).

Fig. 2 compares the effect on asymptomatic, as well as symptomatic, malaria infections of chemicals with different functions: a repellent that only acts to reduce the contact rate between vectors and humans, versus a chemical that incurs mosquito mortality in addition to reducing contact rates. The comparison is made across the qualitatively different biting responses of vectors (Types I–V) and across a range of different alternative host species availability. Invariably, mosquitocidal chemicals were better at controlling infection than repellents. In most scenarios, a small increase in symptomatic infection prevalence resulted from moderate control levels and this came about through a reduced force of infection delaying the progression to the more benign (asymptomatic) state. However, this was more than offset by the reduction in asymptomatically infected individuals and so the total infection prevalence was always reduced by control.

Going across the columns, the intuitive, positive relationship between a higher prevalence of infection for populations that are more human-dominated (thereby fewer bites wasted on incompetent hosts) can clearly be seen. Comparing down the different biting response types also shows several biologically intuitive outcomes. Firstly, an anthropophilic mosquito (Type II) is the best malaria vector giving rise to the highest prevalences and necessitating the highest levels of control. Concordantly, a zoophilic mosquito (Type IV) is the worst malaria vector giving rise to the lowest prevalences and greatest amenability to control. Projections of these extremes in vector biting behaviour relative to the classic assumption (Type I) yielded greater disparity for populations that were increasingly cattle-dominated. Again, this would be expected — when there are fewer options to bite anything but humans, local conditions drive vector biting behaviour and intrinsic preferences are of reduced influence.

**Fig. 3:**
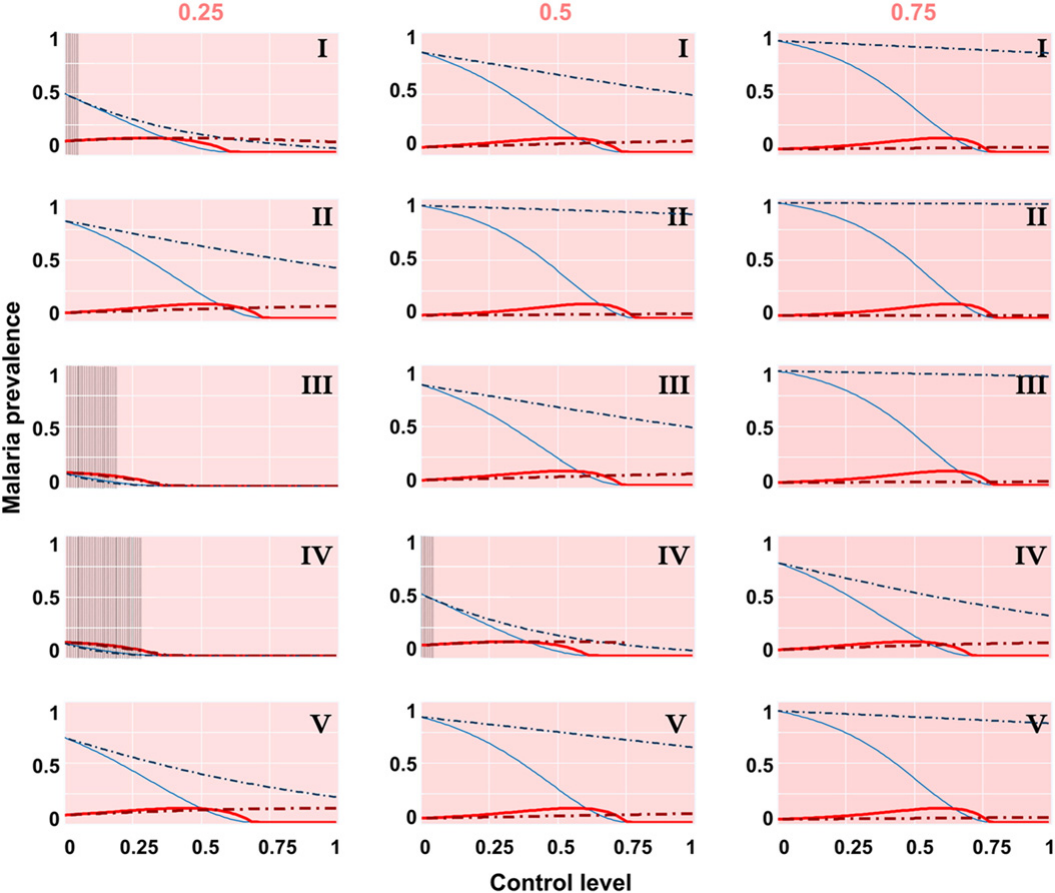
Malaria prevalence (asymptomatic in blue; symptomatic in red) following human-targeted (solid lines) versus cattle treatment (broken lines) with mosquitocidal products. Control efficacy is shown across the qualitatively distinct mosquito biting ecologies (top right of sub-plots) and across a range of the human availability relative to all blood-hosts (top of sub-plot columns). Vertical shading denotes regions whereby cattle treatment reduced malaria prevalence (asymptomatic plus symptomatics) more than human-targeted approaches.

A mosquitocidal product that was either human-targeted or cattle-targeted was compared for its efficacy in controlling malaria infections (Fig. 3). Treating cattle with topical or chemical insecticides had improved efficacy in controlling malaria for populations that were more cattle-dominant (the gradient of decrease in infection was greatest when cattle compose 75% of the available blood-meal source). The maximum level of control achievable when treating cattle was profoundly impacted by vector biting behavioural ecology. Negligible control was achieved using a cattle-targeted approach for a anthropophilic vector (Type II) in a human-dominated area. However, more substantial gains were achieved when cattle matched or exceeded human availability for all behavioural ecologies — including anthropophilic vectors. Complete control was achievable with cattle-targeted insecticides for vectors exhibiting certain behaviours: Types III and IV mosquitoes were sufficiently reduced to interrupt transmission when cattle exceeded human availability. Specific scenarios arose during simulations in which a cattle-targeted approach actually exceeded malaria control efficacy of more traditional human-targeted approaches (shaded regions of parameter space in Fig. 3).

**Fig. 4:**
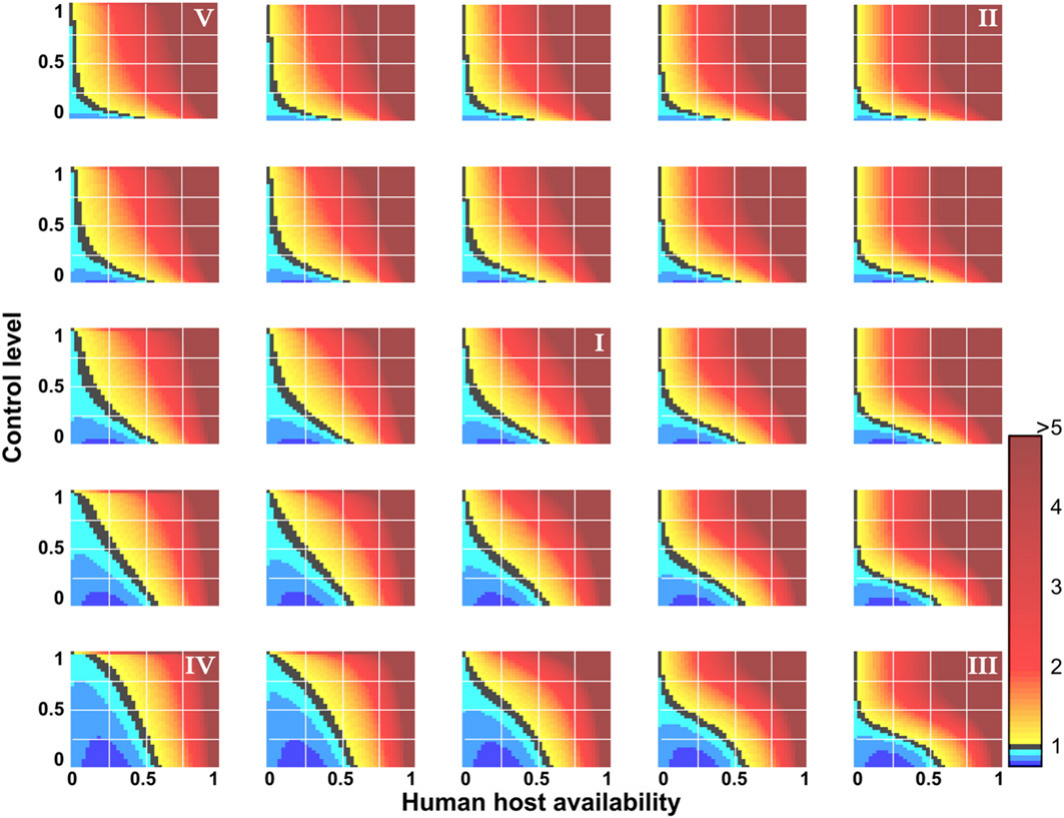
The ratio of effective reproduction numbers following cattle treated with endectocides: human-targeted mosquitocides (colourmap legend in bottom right). The impact of different biting ecologies (top-right of subplots; configuration matches Fig. 1) is shown across the full range of human host availability and control levels.

The effective reproduction numbers were calculated for control using both approaches to determine the combinations of mosquito behaviour types and host availabilities for which a cattle-targeted approach would be advantageous (Fig. 4). For all modelled scenarios, parameter space existed in which a cattle-targeted approach was more effective than human-targeted insecticides. At low human availability (relative to cattle) and when only modest levels of control are achievable, targeting cattle with insecticides was the more effective approach. This parameter space constituted only a small component of most mosquito biting ecologies, with larger regions for zoophilic mosquitoes (Type IV) and those that demonstrated switched preferences towards humans only at low alternative host availabilities (Type III). For the more extreme zoophilic mosquitoes, approaching half the parameter space (of host availability versus control level) was more conducive to control with a cattle-targeted approach. Importantly, however, the improvement in reducing malaria transmission potential was fairly modest (in the order of 10% or 20%) in these regions; whereas, beyond the threshold of equivalent efficacy, more sudden and drastic transitions were seen and a human-targeted approach potentially yielded many-fold improvements.

## 4 Discussion

Models of diseases spread by arthropod vectors have undergone considerable development in recent years. Corresponding with increased biological understanding there has been a shift towards greater model complexity — a trend that has been accommodated by the rapid, contemporaneous improvement in computational power. Examples of complex models with detailed vector elements include ONCHOSIM, a simulation model for the spread of onchocerciasis by the Simulium blackfly (Plaisier et al., 1990); CIMSiM, a container-inhabiting mosquito simulation model (Focks et al., 1993) and associated DenSiM, a dengue simulation model (Focks et al., 1995); and LYMFASIM, a lymphatic filariasis simulation model (Plaisier, A.P., et al., 1998, Stolk, W.A., et al., 2008). Several open-source simulation softwares and platforms currently exist for malaria, and include OpenMalaria (Chitnis et al., 2012), Malaria Tools (Griffin et al., 2010) and the Malaria Model component of EMOD — Epidemiological Modelling software (Eckhoff, 2011).

However, even parameter-rich models have core elements that are identical, or analogous, to the original Ross–Macdonald equation (Reiner et al., 2013), demonstrating the universal foundations of vector-borne disease models. Central to even the most complex of contemporary transmission models is the assumption that the intrinsic host preference of a vector (Bailey, 1982) is fixed. Crucially, a linear functional response (Type I) is without precedent among empirical studies of arthropod resource utilisation (Jeschke et al., 2004).

A new, simple and flexible formula for capturing several qualitatively distinct mosquito biting behaviours is presented. This formula was used to explore the impact of this aspect of disease vector ecology in the context of chemical control approaches that target mosquitoes blood-feeding on humans or cattle. For human-based chemical applications, mosquitocidal products were more effective than repellents. This result is analogous to findings reported by Yakob et al. (2011) and subsequently corroborated by malaria models produced by others (Killeen et al., 2011). However, the level of additional benefit was largely contingent on the proportional representation of humans among all potential blood-source species with more notable difference in relative benefit for more human-dominated areas. Additionally, the effort required to control malaria was profoundly impacted by the behavioural response type. For example, a Type II mosquito required double the control effort compared with a Type IV mosquito to eliminate transmission when cattle were more available than humans. Generating empirical data (starting with semi-field experiments where alternative host species availability can more easily be manipulated) of this neglected behaviour of disease vectors is a matter of increasing priority in light of recent, novel malaria control methods.

A recent study by Poche et al. (2015) has elicited interest in targeting cattle with systemic (or topical) insecticides as a strategy for reducing malaria transmission. Cattle received doses of oral ivermectin, oral eprinomectin, topical eprinomectin or oral fipronil; and, mosquito mortality continued to be significantly increased following up to 3 weeks of treatment (Poche et al., 2015). This is a welcome outcome given the escalating fears over reports of widespread insecticide resistance to current chemical controls that rely chiefly on pyrethroids (Knox et al., 2014). The current study represents the first theoretical exploration of where and when this approach might be anticipated to have the greatest effect in reducing dependence on the current mainstays.

Like all models, necessary simplifications have been made to present outcomes as transparently as possible and these must be considered in any decision-making process. Future work will look into several aspects not included in the current analysis. First, it is increasingly atypical for a single control tool to be wholly relied upon for malaria control and opportunities will be sought to make best use of this novel method as part of an integrated malaria control strategy. This is an area where mathematical models shine because there are so many combinations of the various control tools as to financially preclude empirically testing them all. Models have had considerable utility in exploring synergistic combination strategies as well as identifying when and why certain tools/chemical classes may not make good partners (Yakob, L., et al., 2011, Yakob, L. and Yan, G., 2009). Indeed, some recent randomised controlled trials have shown good agreement with these modelling predictions (Yakob et al., 2013); however, others have not (Lines & Kleinschmidt, 2015). Reasons for substantial differences between successful and unsuccessful applications of integrated control are currently unknown and require re-exploration in the context of the particular vector biting behaviours described by the burgeoning empirical literature and analysed in the current study.

A further avenue of future research will involve relaxing the assumption that all mosquitoes within a given population behave identically. This is particularly important in the context of multiple blood-host species when they have markedly different competence in parasite transmission. At one extreme, a proportion of mosquitoes in a given population will always opt for humans while a proportion will always opt for cattle; at the other extreme, all mosquitoes on average will bite humans a set proportion of times and cattle a set proportion of times. These behaviours have previously been describes as ‘exclusive’ versus ‘mixed’ biting and shown to drastically impact malaria transmission potential (Yakob et al., 2010). In the current context of bites split between humans and cattle, systemic insecticides that reduce longevity of an exclusive mosquito sub-set that is intent on only ever biting cattle will clearly have attenuated effect. Identifying these between-individual differences in mosquito biting behaviour also constitutes an important direction for empirical work.

Future work will also have to look into temporal dynamics of infection. Perhaps the biggest hurdle to endectocides is their current lack of durability; currently optimised doses are only effective for a matter of weeks. In the shorter term, therefore, this approach might only be feasible in unstable or seasonal malaria settings where endectocidal applications can be strategically timed. Optimised timing for epidemic-prone regions and advantageous combination with insecticidal bednets and indoor residual spray constitute necessary directions for model development and are work in progress.
